# Comparison between laser sheaths, femoral approach and rotating mechanical sheaths for lead extraction

**DOI:** 10.1007/s12471-021-01652-w

**Published:** 2021-12-21

**Authors:** F. A. Bracke, N. Rademakers, N. Verberkmoes, M. Van ’t Veer, B. M. van Gelder

**Affiliations:** Department of Cardiology and Cardiothoracic Surgery, Catharina Hospital, Eindhoven, The Netherlands

**Keywords:** Artificial cardiac pacing, Implantable defibrillator, Intraoperative complications, Excimer laser

## Abstract

**Introduction:**

Efficiency and safety are important features in the selection of lead extraction tools. We report our experience with different endovascular techniques to extract individual pacing and defibrillator leads.

**Methods:**

This is a single-centre study of consecutive lead extraction procedures from 1997 until 2019. A total of 1725 leads were extracted in 775 patients. Direct traction sufficed for 588 leads, and 22 leads were primarily removed by surgery. The endovascular techniques used in the remainder were a laser sheath (190 leads), the femoral approach (717 leads) and rotating mechanical sheaths (208 leads).

**Results:**

The three approaches were comparably effective in completely removing the leads (*p* = 0.088). However, there were more major complications with the laser sheath than with the femoral approach or rotating mechanical sheaths (8.4%, 0.5% and 1.2%, respectively). Therefore, the procedural result—extraction without major complications—was significantly better with both the femoral approach and rotating mechanical sheaths than with the laser sheath (*p* < 0.001). This result was confirmed after propensity score matching to compensate for differences between lead cohorts (*p* = 0.007). Cross-over to another endovascular tool was necessary in 7.9%, 7.1% and 8.2% of laser, femoral and rotating mechanical attempts, respectively.

**Conclusion:**

All three endovascular lead extraction techniques showed comparable efficacy. However, there were significantly more major complications using the laser sheath compared to the femoral approach or rotating mechanical sheaths, leading us to abandon the laser technique. Importantly, no single endovascular technique sufficed to successfully extract all leads.

## What’s new?

- No single endovascular extraction tool suffices to obtain optimal results in lead extraction.
- The laser sheath results in significantly more complications than either rotating mechanical sheaths or a femoral approach.
- Laceration of the superior vena cava is the main complication associated with use of the laser sheath.
- A hybrid approach, with the femoral approach as the initial tool for atrial and coronary sinus leads and rotating mechanical sheaths for right ventricular leads, provides the highest efficacy and the lowest complication rate.

## Introduction

With the introduction of laser sheaths in the late 1990s, the speed and success rate of lead extraction greatly improved compared to the previously used plastic sheaths [1, 2]. In 1997, a femoral approach was also developed that used a long sheath and a dedicated snare (Needle’s Eye snare; Cook Medical, Bloomington IN, USA) to catch the lead from the femoral vein. In 2007 a unidirectional rotating mechanical sheath was introduced with cutting blades on the outside of the tip [3]. The next generation, the Evolution RL of Cook Medical, was presented in 2012 with a less aggressive tip and bidirectional rotation to prevent entanglement of the leads. One year later, Spectranetics (Colorado Springs, CO, USA) introduced a comparable sheath.

These tools are still in use today with remarkably similar reported outcomes. However, comparative studies are rare. Therefore, the choice between tools is often determined by operator preference and device cost rather than established superiority. In this report, we compare our experience with different endovascular extraction tools.

## Methods

### Study population

All lead extractions from May 1997 until August 2019 with at least one lead implanted for more than 1 year were included. Patients with a primary surgical extraction procedure or in whom all leads could be extracted with traction alone, either directly or with locking stylets, were excluded.

### Extraction procedure

All procedures were performed during general anaesthesia with the patients prepared for emergency thoracotomy and a surgical team and equipment on standby inside the operating room.

We always tried first to remove the leads using traction with standard or locking stylets. If this failed, our next step evolved over time. At first, we used the Spectranetics laser sheath as our primary extraction tool, with the femoral approach as a backup. We changed to the femoral approach as the primary tool for all pacing leads in 2006, but the laser sheath remained the primary tool for most defibrillator leads. All femoral procedures were performed with a curved Byrd Femoral Workstation sheath and a Needle’s Eye snare (Cook Medical). Finally, in 2013 with the introduction of the Evolution RL (Cook Medical) and Tightrail (Spectranetics) sheaths we switched to the rotating mechanical sheaths as a first-line tool. The femoral approach then remained the first choice for atrial and coronary sinus leads.

### Endpoints

The endpoints are adapted from the 2017 Heart Rhythm Society expert consensus statement [4]. However, we report the success rate on the level of individual leads instead of on a patient level, as different approaches were often used in the same patient. We defined radiological lead success as the removal of the complete lead. For procedural lead success the absence of any permanently disabling complication, procedure-related death, or any unscheduled major surgical intervention (even if followed by full recovery) was added. Clinical lead success is removal of the lead with the possible exception of a small portion (< 4 cm) with similar restrictions.

The incidence of major complications resulting from application of the tools also included those arising from their use as a backup tool. Major complications were those that posed an immediate threat to life or that resulted in death. Minor complications were all undesired adverse events that required medical intervention, including minor procedural interventions, but did not significantly affect the patient’s function.

### Statistical analysis

Continuous values are presented as median and interquartile range and comparisons were performed using the Kruskal-Wallis test. Categorical values are presented as numbers and percentages and comparisons were made using the chi-squared test or Fisher’s exact test as appropriate. A *p*-value of 0.05 was considered statistically significant. All analyses were performed using R statistical software version 4.0.0 (R Foundation for Statistical Computing, Vienna, Austria). Propensity score matching was applied using the MatchIt version 3.0.2 package in R. Propensity scores were determined using nearest-neighbour matching and a caliper value of 0.15.

## Results

### Patients and leads

In the study period, we treated 775 consecutive patients with 1725 leads implanted for more than 1 year. Device infection (89.4%) was the predominant indication (Tab. 1). Traction sufficed for the removal of 33.1% or 588 leads; all leads could be removed with traction in 155 patients. Twenty-two leads (1.3%) were surgically extracted. For the remaining 1115 leads, the initial tool used was a laser sheath in 190, a femoral approach in 717 and rotating mechanical tools in 208 leads (Tab. 2).

**Table 1:** Patient characteristics

**Patients**	
*n*	775
Age (years)^a^	70.3 (61–77.2)
Male	74.5%
ICD	25.4%
CRT	15.5%
Leads per patient^a^	2 (2–3)
Patients with abandoned leads	26.7%
**Indications**	
Infection	89.4%
Pocket infection	55.9%
Systemic infection	16.8%
Endocarditis	16.8%
Non-infectious	10.6%
Lead dysfunction	6.7%
Pain	2.3%
Subclavian vein occlusion	1.0%
Tricuspid regurgitation	0.3%
SVC syndrome	0.1%
Left ventricular lead	0.1%

*SVC* superior vena cava

^a^Median (interquartile range)

**Table 2:** Lead characteristics

		Laser sheath	Femoral approach	RMS	*p*-value
**All leads**	*n* (1115 leads)	190	717	208	
	*Lead characteristics*				
	Implant time (years)^a^	8.1 (4.4–12.0)	7.6 (4.5–11.2)	9.6 (6.5–14.7)	< 0.001
	Location				
	– Atrial	60	328	42	< 0.001
	– Ventricle	130	338	160	
	– Coronary sinus	0	51	6	
	Lead type				
	– Pacing	149	683	148	< 0.001
	– ICD	41	34	60	
	*Success rates per lead*				
	– Radiological	81.6%	87.7%	86.1%	0.088
	– Procedural	75.3%	87.2%	85.1%	< 0.001
	– Clinical	77.4%	90.7%	88.0%	< 0.001
**After propensity score matching**	*n*	160	150	160	
	*Lead characteristics*				
	Implant time (years)^a^	8.7 (4.7–13.2)	7.9 (4.3–12.0)	9.1 (5.8–13)	0.106
	Location				
	– Atrium	40	34	37	0.875
	– Ventricle	120	116	123	
	– Coronary sinus	0	0	0	
	Lead type				
	– Pacing	119	119	121	0.568
	– ICD	41	31	39	
	*Success rates per lead*				
	*n*	160	150	160	
	– Radiological	80.0%	84.0%	88.1%	0.140
	– Procedural	73.8%	84.0%	86.8%	0.007
	– Clinical	75.6%	90.7%	88.8%	< 0.001

*RMS* rotating mechanical sheath

^a^Median (interquartile range)

Implant times were longer in the rotating mechanical sheath group compared to both the femoral approach and laser sheath cohort (*p* < 0.001 for both comparisons). Secondly, there were virtually no coronary sinus leads at the time when we preferred the laser sheath. Thirdly, defibrillator leads were infrequently extracted with a femoral approach because of restraints imposed by the small lumen diameter of the femoral workstation.

### Outcome

The results are shown in Tab. 2. There was no statistical difference in radiological outcome between the three groups (*p* = 0.088). However, procedural and clinical success rates of femoral extraction and rotating mechanical tools were comparable, but those of the laser sheath were significantly lower (*p* < 0.001).

After propensity matching for implant time, the intracardiac location of the lead and lead type, radiological success remained statistically non-significant between the three groups (*p* = 0.140), but procedural and clinical efficacy of the laser sheath were still inferior (*p* = 0.007 and *p* < 0.001, respectively) (Tab. 3).

**Table 3:** Major complications of each extraction technique including their backup use

	Total	Laser sheath	Femoral approach	RMS
*Lead extraction attempts*^*a*^	1188	202	736	250
*Total major complications*	2.0%	8.4%	0.5%	1.2%
*Location of complication:*				
Including extrapericardial SVC	11	10	0	1^c^
Intrapericardial tear	12	6^b^	4	2
LIMA rupture	1	1	0	0
*Mortality (patients)*		7^b^	0	0

*RMS* rotating mechanical sheath, *SVC* superior vena cava, *LIMA* left internal mammary artery

^a^Also including the leads for which a specific technique was used as a backup tool

^b^Including one late tamponade possibly attributed to a temporary pacing wire

^c^Complication of a first-generation Evolution device

Cross-over to another endovascular tool was similar for laser, femoral and rotating mechanical tools: 7.9%, 7.1% and 8.2%, respectively. The combined clinical success per patient of all endovascular extraction tools combined for all leads in a single procedure during the laser sheath, femoral approach and rotating mechanical sheath period was 95.1% in 184 patients, 96.3% in 321 patients and 98.1% in 270 patients, respectively, with major complications in 9.8%, 1.3% and 0.7% of patients, respectively.

### Complications

The laser sheath had a significantly higher complication rate (8.4%) than the other two techniques (Tab. 3). Ten of the eleven complications involving the extrapericardial portion of the superior vena cava resulted from laser sheath procedures. All four major complications of the femoral approach resulted from countertraction whilst extracting right atrial leads (0.5%). There were three complications with rotating mechanical sheaths (1.2%). The only extrapericardial superior vena cava tear in this group was caused by one of the three unidirectional Evolution sheaths we used. With the newer Evolution RL and Tightrail devices we encountered one right atrial tear plus a localised pericardial effusion resulting from the forceful traction needed to pull back a trapped sheath.

Twenty-one of the 24 patients with major complications were immediately operated upon. The exceptions were one patient with limited pericardial effusion who had successful pericardial drainage, a second patient with late tamponade the night after the extraction procedure who remained hypotensive in spite of successful pericardial drainage, and a third patient in whom the left internal mammary artery was severed, causing an arteriovenous fistula needing elective surgical correction.

## Discussion

A first observation is that no single endovascular device sufficed to extract every lead. Therefore, the availability of more than one extraction tool is mandatory. Secondly, use of the laser sheath caused significantly more complications, resulting in inferior procedural and clinical outcomes. There was no statistical difference in outcome and complications between the femoral approach and rotating mechanical sheaths.

The reported efficacy of the three approaches is often quite similar, but there are only limited direct comparisons. The Plexus study is the only randomised trial comparing laser sheaths and plastic telescoping sheaths in 301 procedures [2]. The procedural outcome was in favour of the laser sheath (94% vs 64% complete removal), but with a high level of cross-over indicating an eagerness to use the laser rather than the more laborious plastic sheaths. Major complications occurred in three patients in the laser sheath group versus none with the plastic sheaths. Bordachar et al. randomly assigned 101 patients to either a femoral approach or laser extraction. Procedural success was 88% in both groups [5]. Two patients in the laser sheath and one in the femoral group had a major complication. The authors also compared the results of three centres using the laser sheath (218 patients) with three centres using a femoral approach (138 patients). Procedural success was reported in 85% of laser sheath cases and 86% of femoral procedures. There were two deaths in the laser sheath group and one in the femoral group.

Mazzone et al. reported a single-centre comparison of the laser sheath in 73 patients with the unidirectional Evolution sheath in 48 patients without a statistically significant difference in procedural success (97.3% vs 91.7%) or in major complications (2.7% vs 4.2%) [6]. Starck et al. compared 39 leads extracted with laser sheaths with 99 leads extracted with mechanical sheaths, also with no significant differences in outcome and complications [7].

In the European Electra lead extraction registry of 3510 procedures, there were more major complications and less favourable clinical outcomes with powered sheaths and a femoral approach compared to the plastic telescoping sheaths [8]. Further, the femoral approach performed worse than the powered sheaths. However, no data on the subgroups were available. The same limitations apply to a recent Maude database search which suggested a 4.3 to 19.5 times increased risk of death with laser sheaths compared to rotating sheaths [9].

We experienced a high complication rate with the laser sheath with laceration of the superior vena cava as the major contributor. In contrast, the Lexicon registry of 2405 laser lead extractions reported major complications in only 1.4% of patients with a 0.28% mortality rate [10]. However, these results are not universal. Gaca et al. reported 7 major complications in 112 procedures, and Wang et al. 5% in 140 cases, including 3.6% vascular ruptures with the laser technique [11, 12]. Our complication rate may have resulted from inexperience early on in our practice, although complications peaked only in the final period of our laser use. Further, there was an abrupt drop in complications once we switched to rotating mechanical tools. An explanation for the laser sheath complications may be that its effect is not confined to the direct photochemical vapourisation of cellular structures, but includes explosive photothermal vapourisation of cellular water which produces rapidly expanding bubbles [13, 14]. As a certain contact force is necessary to be effective, the pressurised bubbles trapped in front of the sheath may damage the adjacent vasculature.

With the unidirectional Evolution sheath, with cutting blades directed outwards, we also encountered a vascular tear after prolonged local application in one of three attempts. However, Delnoy et al. performed 54 procedures with the original Evolution sheath, without major complications [15].

In contrast, the Evolution RL or Tightrail sheaths allowed prolonged application at sites of dense scar tissue without vascular lacerations. These sheaths have a less aggressive design with a more forward-directed action, resulting in better protection of the adjacent vasculature. Similar to our experience, Mazzone et al. achieved 91.6% procedural success with the Evolution RL sheath extracting 238 leads in 124 consecutive patients without major complications [16]. Aytemir et al. extracted 42 leads with the Tightrail device with a 95.7% success rate and no complications [17].

There were no venous complications with the femoral approach, as it is not actively used in the upper thoracic veins [18]. However, there was a higher failure rate when extracting right ventricular leads with long implant times [18].

We now use a hybrid approach, with the newer-generation rotating mechanical sheaths as the first-line tool for right ventricular leads and the femoral approach to extract atrial and coronary sinus leads because of both its safety and effectiveness. It should be noted that the femoral approach is technically more demanding [18].

### Limitations

The choice of tools was not randomised but the result of availability and ongoing experience. The latter might have benefited later procedures regardless of the technique. The efficacy of individual techniques is possibly underrated, as we regularly switched to an alternative technique once we encountered insufficient progress or considered that persevering with the attempt was too risky. There was a selection bias, as the femoral approach often remained our initial method to extract atrial and coronary sinus during the later phase of the study.

## Conclusion

No single technique sufficed to successfully extract all leads, stressing the need to have different tools available. The higher complication rate with the laser sheath in our experience negatively influenced the procedural and clinical outcome compared to those of rotating mechanical tools and the femoral approach and was therefore abandoned. Rotating mechanical sheaths have become first choice for right ventricular leads and the femoral approach for most atrial and coronary sinus leads.
